# Positive Islet Cell Cytoplasmic Antibody and Long-Term Use of Lipid-Lowering Agents Are Positively Correlated With Peripheral Atherosclerosis in Patients With Autoimmune Diabetes: A Cross-Sectional Study

**DOI:** 10.1155/jdr/1933825

**Published:** 2025-01-24

**Authors:** Xinyue Chen, Jie Yu, Yiwen Liu, Xuechen Wang, Fan Ping, Wei Li, Huabing Zhang, Lingling Xu, Yuxiu Li

**Affiliations:** Department of Endocrinology, Key Laboratory of Endocrinology, Ministry of Health, Peking Union Medical College Hospital, Peking Union Medical College, Chinese Academy of Medical Sciences, Beijing, China

**Keywords:** autoantibodies, autoimmune diabetes, dyslipidemia, peripheral atherosclerosis

## Abstract

**Aims:** This cross-sectional study is aimed at determining whether systemic inflammation, diabetic autoantibodies, and islet *β* cell dysfunction play a role in the progression of macrovascular complications in patients with autoimmune diabetes.

**Methods:** 202 patients with autoimmune diabetes aged ≥ 35 years and hospitalized in Peking Union Medical College Hospital were enrolled in this study. The patients were divided into three groups based on the severity of peripheral atherosclerosis. Biomarkers of systemic inflammation, diabetes autoantibodies, islet *β* cell function, and other covariates validated to be associated with macrovascular complications were collected. Correlations between the severity of peripheral atherosclerosis and systemic inflammation, diabetic autoantibodies, and islet β cell function were examined using an ordinal logistic regression model.

**Results:** Of the enrolled patients, 39.1% were male, with a median age of 53 (43, 60) years and a diabetes duration of 96 (36, 216) months. 58 patients had no lesions in the peripheral arteries, 72 had atherosclerosis in the carotid or lower extremity arteries, and the rest had lesions in both arteries. In the multifactor ordinal logistic regression test, positive islet cell cytoplasmic antibody (ICA) and long-term use of lipid-lowering agents were independently associated with peripheral atherosclerosis after adjusting for age and diabetes duration.

**Conclusions:** The correlation between positive ICA and atherosclerosis suggests inflammation at an early stage plays a role in macrovascular complications in autoimmune diabetes. The association between long-term use of lipid-lowering agents and atherosclerosis suggests the need for early screening and intervention for dyslipidemia in patients with autoimmune diabetes.

## 1. Introduction

Autoimmune diabetes includes type 1 diabetes mellitus (T1DM) and latent autoimmune diabetes in adults (LADA). The disease is characterized by positive diabetic autoantibodies and islet *β* cell dysfunction, with autoimmunity and inflammation playing a vital role in the pathogenesis [1, 2]. The widespread utilization of insulin, particularly multiple injections and insulin pumps, has significantly extended the life expectancy of patients [1, 3, 4], accompanied by a rapidly increasing incidence of chronic diabetic complications [5–7]. Recent studies have indicated that the morbidity of macrovascular complications in patients with long-term autoimmune diabetes increases steadily and is higher than that in type 2 diabetes, given the longer duration of hyperglycemia and more drastic fluctuations in blood glucose [7–9], making cardiovascular disease the main cause of death in patients with autoimmune diabetes [10].

Recently, researchers have explored risk factors for macrovascular complications in patients with autoimmune diabetes and found that suboptimal glucose control, dyslipidemia, and diabetic microvascular complications promote the development of diabetic macrovascular complications of autoimmune diabetes [11–13]. Previous studies have shown that autoantibodies and inflammatory markers play a role in peripheral atherosclerosis in various autoimmune diseases, such as systemic lupus erythematosus [14]. However, whether autoimmunity and rapid decline in islet *β* cell function are involved in the pathogenesis of macrovascular complications of autoimmune diabetes remains unknown. This single-center cross-sectional study was conducted in Chinese patients with autoimmune diabetes to determine whether autoimmunity and decline in islet *β* cell function play a role in the progression of macrovascular complications in addition to classic risk factors.

## 2. Material and Methods

### 2.1. Cohort Enrollment

**Figure 1:**
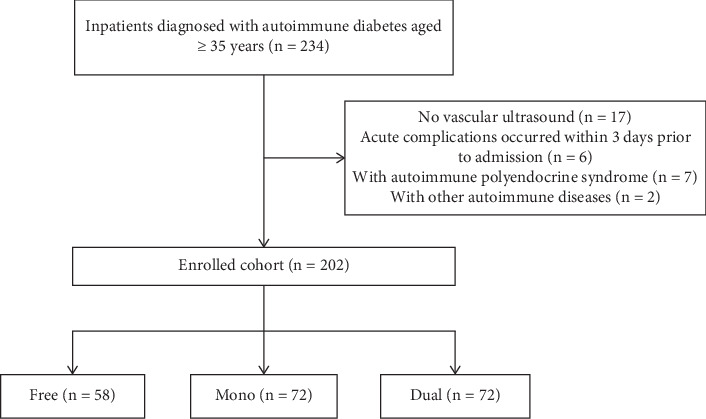
Study flowchart showing inclusion and exclusion criteria. Note: the free group refers to patients without lesions in either the carotid or lower extremity arteries; the mono group refers to patients with lesions in either the carotid or lower extremity arteries; the dual group refers to patients with lesions in both the carotid and lower extremity arteries.

Patients with T1DM or LADA discharged from the endocrine ward of Peking Union Medical College Hospital (PUMCH) between January 2016 and June 2022 and who met the following criteria were enrolled: (1) aged ≥ 35 years and (2) underwent ultrasound examination of the carotid and lower extremity arteries during hospitalization (using a LOGIQ E9-138 ultrasound system (GE Healthcare, Waukesha, Wisconsin, United States)). The exclusion criteria were patients with (1) acute diabetic complications that occurred within 3 days before admission, (2) autoimmune polyendocrine syndrome, and (3) other autoimmune diseases such as vasculitis. The final cohort comprised 202 patients (Figure 1). The data analyzed in this study were extracted from the PUMCH Electronic Medical Records Analytical Database (PUMCH-EMERALD). Personal information such as name and ID number was not included in the study. Thus, the study was approved by the Ethics Committee of PUMCH without the need for written informed consent (Approval Number: K3897-K23C1812).

The cohort was stratified into three groups according to the severity of peripheral atherosclerosis by ultrasound examination [15]: group free (no lesions in both carotid and lower extremity arteries), group mono (lesions in carotid or lower extremity arteries), and group dual (lesions in both carotid and lower extremity arteries). Lesions were defined as the presence of intimamedia thickness > 0.9 mm or established plaque [16].

### 2.2. Clinical Characteristic Collection

#### 2.2.1. Sociodemographic, Previous Medical History, and Anthropometric Data

Data on sociodemographic characteristics (age, gender, and autoimmune diabetes duration (DURATION)), medical history (long-term use of lipid-lowering agents), lifestyle factors (smoking history and alcohol intake history), and anthropometric parameters (heart rate (HR), systolic blood pressure (SBP), diastolic blood pressure (DBP), body mass index (BMI), and waist circumference (WC)) were extracted from admission records of the PUMCH-EMERALD.

#### 2.2.2. Laboratory Measurements

Laboratory parameters related to the following aspects were included in this study for analysis: (1) parameters of systemic inflammation (white blood cell counts (WBC), neutrophil/lymphocyte ratio (NLR), and C reactive protein (CRP)); (2) whether diabetic autoantibodies (glutamic acid decarboxylase autoantibody (anti-GAD), antityrosine phosphatase antibody (anti-IA2), and islet cell cytoplasmic autoantibody (ICA)) were positive; (3) biomarkers reflecting islet *β* cell function (fasting C-peptide (CP-0h) and 2h proprandial C-peptide (CP-2h)); (4) other covariates related to atherosclerosis, including glucose metabolism parameters (fasting blood glucose (FBG), 2h-postprandial blood glucose (2h-PBG), and glycated hemoglobin (HbA1c)), lipid profile status (total cholesterol (TC), triglyceride (TG), high-density lipoprotein cholesterol (HDL-c), and low-density lipoprotein cholesterol (LDL-c)), and indicators of diabetic nephropathy (estimated glomerular filtration rate (eGFR) calculated using the Cockcroft–Gault formula ((140 − age) × weight (kg) × 88.4/(72 × serum creatinine concentration (Scr (*μ*mol/L))) and urinary albumin creatinine ratio (ACR)].

Among the biomarkers mentioned above, diabetic autoantibodies were analyzed by an automated chemistry analyzer (SMART 6500 HOB, Keysmile Biological Technology Co., China) using the chemiluminescence method. CP was analyzed with an automated chemistry analyzer (Siemens Centaur XP, Siemens AG, Germany) using the chemiluminescence method. CRP, blood glucose, lipid parameters, and SCr levels were assessed using an automated chemistry analyzer (Beckman AU, Beckman Coulter Inc., United States). HbA1c level was measured using high-performance liquid chromatography (BIO-RAD D-100, United States). WBC, neutrophil count, and lymphocyte count were automatically measured using a Siemens ADVIA2120 or Sysmex XN9100 analyzer (Siemens, Munich, Germany; Sysmex America, Illinois, United States).

### 2.3. Statistical Analysis

First, the continuous variables were subjected to a normality test. Normally distributed variables are presented as means and standard deviations (SDs), with analysis of variance (ANOVA) used for comparison, and nonnormally distributed variables are presented as interquartile ranges, with the Kruskal−Wallis test used for comparison. Categorical variables are presented as frequencies, and the chi-square test revealed significant differences. A bivariate correlation test between potential risk factors and the severity of peripheral atherosclerosis was performed using Spearman's test. Furthermore, the false discovery rate (FDR) method was used in the ANOVA, Kruskal−Wallis, chi-square, and Spearman's tests to adjust for multiple test problems. Finally, ordinal logistic regression model was used to determine whether diabetic autoantibodies, systemic inflammation biomarkers, or C-peptide levels were independently correlated with the severity of peripheral atherosclerosis. All *p* values were two-sided and considered significant at 0.05. Statistical analyses were carried out using SPSS 26.0 and R 4.4.0.

## 3. Results

A total of 202 patients (39.1% male, median age of 53 (43.60) years, and DURATION of 96 (36.216) months) were included and further stratified into three groups: The free group comprised 58 patients without detectable lesions; the mono group included 72 patients with atherosclerosis in either the carotid arteries or lower extremity arteries, and the dual group comprised the remaining 72 participants with atherosclerosis in both the carotid arteries and lower extremities arteries.

### 3.1. Association Between Peripheral Atherosclerosis and Clinical and Laboratory Characteristics

Table 1 compares the parameters according to the severity of peripheral atherosclerosis. Compared with the other groups, patients in the dual group tended to be older with a lower eGFR. Additionally, the rate of long-term use of lipid-lowering agents was higher in the dual group than in the other groups. Meanwhile, patients in the dual group also had a longer disease duration and higher SBP, NLR, and HbA1c levels, with a greater possibility of positive ICA; however, these differences were not significant after adjustment using the FDR test.

### 3.2. Positive ICA Was Independently Positively Associated With Peripheral Atherosclerosis in Patients With Autoimmune Diabetes Apart From Age and Long-Term Use of Lipid-Lowering Agents

The coefficient correlations between the potential risk factors and the severity of peripheral atherosclerosis are detailed in Table 2. Positive correlations were identified between the severity of peripheral atherosclerosis and age, DURATION, long-term use of lipid-lowering agents, SBP, and HbA1c. Peripheral atherosclerosis was negatively correlated with eGFR. Additionally, the NLR, CP-0, CP-2h, positive ICA, and ACR tended to be correlated with the severity of peripheral atherosclerosis, which did not reach significance after adjusting for multiple tests.

Parameters with adjusted *p* value below 0.2 in Spearman's test were further assessed in a multifactor ordinal logistic regression (Table 3). Apart from long-term use of lipid-lowering agents and age, positive ICA was independently positively associated with peripheral atherosclerosis (OR = 2.737, 95% CI: 1.215–6.165).

## 4. Discussion

In this single-center cross-sectional study of adult Chinese patients with autoimmune diabetes, we found that positive ICA was independently and positively associated with macrovascular complications, which is a novel finding in the research on macrovascular complications of autoimmune diabetes. Furthermore, the long-term use of lipid-lowering agents was confirmed to be associated with macrovascular complications in Chinese patients with autoimmune diabetes. However, C-peptide levels have not been found to be correlated with atherosclerosis.

Our study contributes to a notable gap, as the existing research on the relationship between autoantibodies and atherosclerosis in autoimmune diabetes is limited. We found a robust relationship between ICA and the manifestations of peripheral atherosclerosis. Previous studies have highlighted the significance of diabetic autoantibodies as indicators of the rapid development of autoimmune diabetes [13, 17]. Another cross-sectional study showed that more positive autoimmune diabetes-associated antibodies are associated with a lower frequency of metabolic syndrome, which is a risk factor for peripheral atherosclerosis [18]. Combining the results of previous studies with those of our study suggests that a positive ICA may not affect atherosclerosis by aggravating abnormal glucose and lipid metabolism. Furthermore, the NLR appears to be associated with peripheral atherosclerosis; therefore, whether ICA-mediated inflammation plays a role requires further investigation. Research on the progression of atherosclerosis has indicated that ascending levels of inflammatory factors such as CRP and interleukin 6 play a role in chronic inflammation during the process of atherosclerosis formation [19, 20]. An earlier study in a small cohort suggested a positive relationship between CRP and positive ICA [21], which was not found in our study and remains controversial [22]. Current evidence suggests that ICA may promote the occurrence of chronic inflammation by influencing the levels of inflammatory factors and further lead to the development of diabetic macrovascular complications. The findings of this study underscore the need for prospective research to confirm the enduring impact of baseline autoantibodies on the progression of peripheral atherosclerosis and its potential mechanisms.

In the past, studies on the effects of residual C-peptide levels in patients with T1DM have mainly focused on its effects on microvascular complications and neuropathy. A review of previous studies revealed that residual C-peptide levels were negatively correlated with the risk of diabetic nephropathy, retinopathy, and neuropathy [23], and even if peak C-peptide levels are reduced to ≥ 0.2 nmol/L, the risk of chronic complications can be reduced compared with the complete loss of islet β cell function [24]. It can be inferred from the above that the retention of C-peptide levels is conducive to delaying the development of atherosclerosis as a parallel relationship between macrovascular and microvascular complications. Regarding the mechanism, on one hand, it is believed that the preserved islet function represented by C-peptide plays a role in improving the state of glucose and lipid metabolism and thus delaying the development of cardiovascular diseases [25]. In contrast, C-peptide has recently been shown to be biologically active, delaying the progression of cardiovascular disease in patients with T1DM by reducing oxidative stress levels, reducing the toxicity of hyperglycemia, and inhibiting vascular smooth muscle proliferation [26]. Additionally, decreased C-peptide levels affect the mobilization of endothelial progenitor cells and vascular function, which may also be involved in its prevention [27]. In this study, no correlation was found between C-peptide levels and the severity of peripheral atherosclerosis. The reason for these different results may be that this was a cross-sectional study that included patients with generally low C-peptide levels. Future prospective clinical studies with larger sample sizes are required to confirm the role of the residual islet β cell function in delaying the occurrence and development of diabetic macrovascular complications.

In our study, we found no significant differences in serum lipid levels among different severities of peripheral atherosclerosis, partly in line with the abnormal characteristics of lipid metabolism in autoimmune diabetes, which are weaker than those in type 2 diabetes [28, 29]. We also explored the relationship between long-term use of lipid-lowering agents, another indicator of abnormal lipid metabolism, and peripheral atherosclerosis and found an independent positive correlation. A previous large-scale prospective study suggested that long-term use of lipid-lowering drugs such as statins can significantly reduce the risk of acute cardiovascular events in patients with T1DM [30]. The reason for not reaching the same conclusion in this study is that it was a cross-sectional study and failed to demonstrate the longitudinal effects of long-term lipid-lowering drugs on peripheral atherosclerosis. Additionally, the current lipid reduction targets for autoimmune diabetes are as intensive as those for type 2 diabetes [31, 32]. Lipid-lowering agents play an important role in lowering blood lipid levels and protecting patients with autoimmune diabetes from macrovascular complications [10]. The protective effect against atherosclerosis and the formulation of lipid-lowering goals for patients with autoimmune diabetes need to be validated in prospective studies in Chinese patients with autoimmune diabetes without atherosclerosis.

The influence of systemic inflammation and islet *β* cell function on atherosclerosis in autoimmune diabetes, explored in this study, remains a novel field. However, this study has several limitations that should be considered. First, the study used a relatively small sample size. Second, as a cross-sectional study, the results of the coefficient correlation test were suboptimally validated than longitudinal studies and failed to establish causal relationships. Third, as continuous glucose monitoring data were lacking, the influence of daily fluctuations in blood glucose on peripheral atherosclerosis was not considered in this study. Fourth, there was no control group of patients with or without type 2 diabetes, making the results less comprehensive. Further studies should include larger and more diverse samples and focus on both in and outpatients to explore the risk factors for atherosclerosis in autoimmune diabetes and the potential mechanisms.

## 5. Conclusions

This cross-sectional study explored the risk factors for peripheral atherosclerosis in adult Chinese patients with autoimmune diabetes. The presence of diabetic autoantibodies, especially ICA, was independently and positively associated with peripheral atherosclerosis. Additionally, long-term use of lipid-lowering agents was positively associated with the occurrence of peripheral atherosclerosis. Our findings suggest that early screening of serum lipid levels and peripheral atherosclerosis in patients with autoimmune diabetes, coupled with timely intervention for lipid-lowering and inhibition of inflammation, may effectively prevent diabetic macrovascular complications at an early stage.

## Figures and Tables

**Table 1:** Comparison of clinical parameters among the groups with different severities of atherosclerosis.

**Variable**	**Free (** **n** = 58**)**	**Mono (** **n** = 72**)**	**Dual (** **n** = 72**)**	**Adjusted ** **p** ** value**
Age (years)	44.5 (40, 54)	50.5 (44, 47)	62 (54.25, 67)	< 0.001^###^
Male	37.90% (22)	37.50% (27)	41.70% (30)	0.856
Duration (months)	54 (15, 144)	103.5 (45, 216)	132 (39.5, 240)	0.074
Smoking history	32.8% (19)	22.2% (16)	33.3% (24)	0.589
Alcohol intake history	32.8% (19)	22.2% (16)	26.4% (19)	0.589
Long-term use of lipid-lowering agents	15.5% 7(9)	25% (18)	44.4% (32)	0.007^##^
SBP (mmHg)	120 (108, 130.5)	124.5 (113, 135.75)	128.5 (117, 144)	0.083
DBP (mmHg)	73.83 ± 11.38	75.72 ± 11.49	74.26 ± 10.59	0.831
HR (bpm)	78 (72, 84)	80 (76, 85.75)	78 (73.25, 86)	0.483
BMI (kg/m^2^)	22.89 (21.02, 24.93)	22.19 (20.77, 24.55)	22.00 (19.99, 24.90)	0.762
WC (cm)	81.5 (74.75, 88)	82 (73.2, 88.5)	83 (75, 91)	0.762
WBC (×10^9^/L)	4.89 (4.06, 6.07)	4.98 (4.15, 6.35)	5.5 (4.29, 6.25)	0.611
NLR	1.795 (1.27, 2.16)	1.99 (1.4925, 2.4925)	2.025 (1.595, 2.5225)	0.183
CRP (mg/L)	0.72 (0.36, 1.52)	0.6 (0.34, 1.10)	0.61 (0.38, 2.31)	0.762
CP-0 (ng/mL)	0.05 (0.03, 0.12)	0.06 (0.03, 0.27)	0.08 (0.03, 0.22)	0.373
CP-2h (ng/mL)	0.05 (0.03, 0.17)	0.07 (0.03, 0.31)	0.08 (0.03, 0.45)	0.341
Positive anti-GAD	35.70% (20/56)	44.10% (30/68)	37.30% (25/67)	0.682
Positive anti-IA2	11.50% (6/52)	20.60% (14/68)	16.90% (11/65)	0.589
Positive ICA	12.50% (7/56)	25.40% (16/63)	32.30% (20/62)	0.137
FBG (mmol/L)	9.9 (7.23, 13.48)	9.1 (6.33, 14.53)	9.6 (6.93, 12.75)	0.978
2h-PBG (mmol/L)	13.4 (9.43,18.53)	13.5 (8.2, 18.4)	14.6 (9.7, 18.25)	0.999
HbA1c (%)	7.8 (6.98, 8.73)	8.2 (7.2, 9.53)	8.75 (7.7, 9.5)	0.074
eGFR (mL/min)	93.48 ± 18.63	86.14 ± 22.00	76.17 ± 25.17	< 0.001^###^
ACR (mg/g Cr)	6 (3, 17)	5 (3, 18)	8 (4, 26.75)	0.341
TC (mmol/L)	4.515 (3.88, 5.38)	4.68 (3.85, 5.40)	4.59 (3.91, 5.10)	0.992
TG (mmol/L)	0.695 (0.49, 0.89)	0.75 (0.57, 1.13)	0.75 (0.56, 1.15)	0.598
HDL-c (mmol/L)	1.56 ± 0.43	1.49 ± 0.43	1.49 ± 0.44	0.762
LDL-c (mmol/L)	2.50 (1.96, 2.94)	2.43 (1.90, 3.12)	2.45 (1.79, 3.08)	0.992

*Note:* Due to the missing values for the diabetes autoantibody test, the actual number was marked as (number of positive cases/total cases).

Abbreviations: 2h-PBG, 2-h postprandial blood glucose; ACR, urinary albumin-to-creatinine ratio; anti-GAD, glutamic acid decarboxylase antibody; anti-IA2, antityrosine phosphatase antibody; BMI, body mass index; CP-0, fasting C-peptide; CP-2h, 2-h postprandial C-peptide; CRP, C-reactive protein; DBP, diastolic blood pressure; eGFR, estimated glomerular filtration rate; FBG, fasting blood glucose; HbA1c, glycated hemoglobin; HDL-c, high-density lipoprotein cholesterol; HR, heart rate; ICA, islet cell cytoplasmic antibody; LDL-c, low-density lipoprotein cholesterol; NLR, neutrophil/lymphocyte ratio; SBP, systolic blood pressure; TC, total cholesterol; TG, triglyceride; WBC, white blood cell count; WC, waist circumference.

^##^ *p* value < 0.01.

^###^ *p* value < 0.001.

**Table 2:** Coefficient correlations between clinical parameters and severity of peripheral atherosclerosis.

**Variable**	**Correlation coefficient with peripheral atherosclerosis**	**Adjusted ** **p** ** value**	**Variable**	**Correlation coefficient with peripheral atherosclerosis**	**Adjusted ** **p** ** value**
Age (years)	0.557	< 0.001^###^	CP-0 (ng/mL)	0.136	0.157
Male	0.033	0.849	CP-2h (ng/mL)	0.143	0.156
Duration (months)	0.204	0.021^#^	Positive anti-GAD	0.007	0.954
Smoking history	0.016	0.884	Positive anti-IA2	0.049	0.775
Alcohol intake history	−0.050	0.774	Positive ICA	0.186	0.051
Long-term use of lipid-lowering agents	0.259	0.002^##^	FBG (mmol/L)	−0.029	0.858
HR (bpm)	0.041	0.821	2h-PBG (mmol/L)	−0.001	0.984
SBP (mmHg)	0.198	0.023^#^	HbA1c (%)	0.208	0.021^#^
DBP (mmHg)	−0.022	0.858	eGFR (mL/min)	−0.307	<0.001^###^
BMI (kg/m^2^)	−0.076	0.511	ACR (mg/g Cr)	0.127	0.195
WC (cm)	0.079	0.511	TC (mmol/L)	−0.023	0.858
WBC (×10^9^/L)	0.099	0.389	TG (mmol/L)	0.088	0.445
NLR	0.154	0.102	HDL-c (mmol/L)	−0.069	0.568
CRP (mg/L)	0.039	0.829	LDL-c (mmol/L)	−0.021	0.858

Abbreviations: 2h-PBG, 2-h postprandial blood glucose; ACR, urinary albumin-to-creatinine ratio; anti-GAD, glutamic acid decarboxylase antibody; anti-IA2, antityrosine phosphatase antibody; BMI, body mass index; CP-0, fasting C-peptide; CP-2h, 2-h postprandial C-peptide; CRP, C-reactive protein; DBP, diastolic blood pressure; eGFR, estimated glomerular filtration rate; FBG, fasting blood glucose; HbA1c, glycated hemoglobin; HDL-c, high-density lipoprotein cholesterol; HR, heart rate; ICA, islet cell cytoplasmic antibody; LDL-c, low-density lipoprotein cholesterol; NLR, neutrophil/lymphocyte ratio; SBP, systolic blood pressure; TC, total cholesterol; TG, triglyceride; WBC, white blood cell count; WC, waist circumference.

^#^ *p* < 0.05.

^##^ *p* < 0.01.

^###^ *p* < 0.001.

**Table 3:** Odds ratios and 95% CIs in multifactor ordinal logistic regression for risk factors for peripheral atherosclerosis.

	**OR (95% CI)**	**p** ** value**
Age (years)	1.126 (1.077, 1.177)	< 0.001^###^
Duration (months)	1.001 (0.998, 1.004)	0.535
Long-term use of lipid-lowering agents	2.298 (1.090, 4.845)	0.029^#^
SBP (mmHg)	1.009 (0.990, 1.028)	0.361
NLR	1.282 (0.900, 1.829)	0.168
CP-0 (ng/mL)	1.151 (0.123, 10.805)	0.902
CP-2h (ng/mL)	0.885 (0.317, 2.469)	0.815
Positive ICA	2.737 (1.215, 6.165)	0.015^#^
HbA1c (%)	1.108 (0.892, 1.378)	0.353
eGFR (mL/min)	1.001 (0.983, 1.020)	0.893
ACR (mg/g Cr)	1.000 (0.999, 1.001)	0.055

Abbreviations: ACR, urinary albumin-to creatinine ratio; CI, confidence interval; CP-0, fasting C-peptide; CP-2h, 2-h postprandial C-peptide; eGFR, estimated glomerular filtration rate; HbA1c, glycated hemoglobin; ICA, islet cell cytoplasmic autoantibody; NLR, neutrophil/lymphocyte ratio; SBP, systolic blood pressure.

^###^ *p* < 0.001.

^#^ *p* < 0.05.

## Data Availability

The datasets supporting the conclusions of this article are included in the main text including the model description. All other datasets used and analyzed in the current study are available from the corresponding authors upon request.
